# Hydrogel contained valproic acid accelerates bone-defect repair via activating Notch signaling pathway in ovariectomized rats

**DOI:** 10.1007/s10856-021-06627-2

**Published:** 2021-12-23

**Authors:** Zhou-Shan Tao, Tian-Lin Li, Hong-Guang Xu, Min Yang

**Affiliations:** Department of Trauma Orthopedics, The First Affiliated Hospital of Wannan Medical College, Yijishan Hospital, No. 2, Zhe shan Xi Road, Wuhu, 241001 Anhui People’s Republic of China

## Abstract

The purpose was to observe whether valproic acid (VPA) has a positive effect on bone-defect repair via activating the Notch signaling pathway in an OVX rat model. The MC3T3-E1 cells were cocultured with VPA and induced to osteogenesis, and the osteogenic activity was observed by alkaline phosphatase (ALP) staining, Alizarin Red (RES) staining and Western blotting (WB). Then the hydrogel-containing VPA was implanted into the femoral epiphysis bone-defect model of ovariectomized (OVX) rats for 12 weeks. Micro-CT, biomechanical testing, histology, immunofluorescence, RT-qPCR, and WB analysis were used to observe the therapeutic effect and explore the possible mechanism. ALP and ARS staining and WB results show that the cell mineralization, osteogenic activity, and protein expression of ALP, OPN, RUNX-2, OC, Notch 1, HES1, HEY1, and JAG1 of VPA group is significantly higher than the control group. Micro-CT, biomechanical testing, histology, immunofluorescence, and RT-qPCR evaluation show that group VPA presented the stronger effect on bone strength, bone regeneration, bone mineralization, higher expression of VEGFA, BMP-2, ALP, OPN, RUNX-2, OC, Notch 1, HES1, HEY1, and JAG1 of VPA when compared with OVX group. Our current study demonstrated that local treatment with VPA could stimulate repair of femoral condyle defects, and these effects may be achieved by activating Notch signaling pathway and acceleration of blood vessel and bone formation.

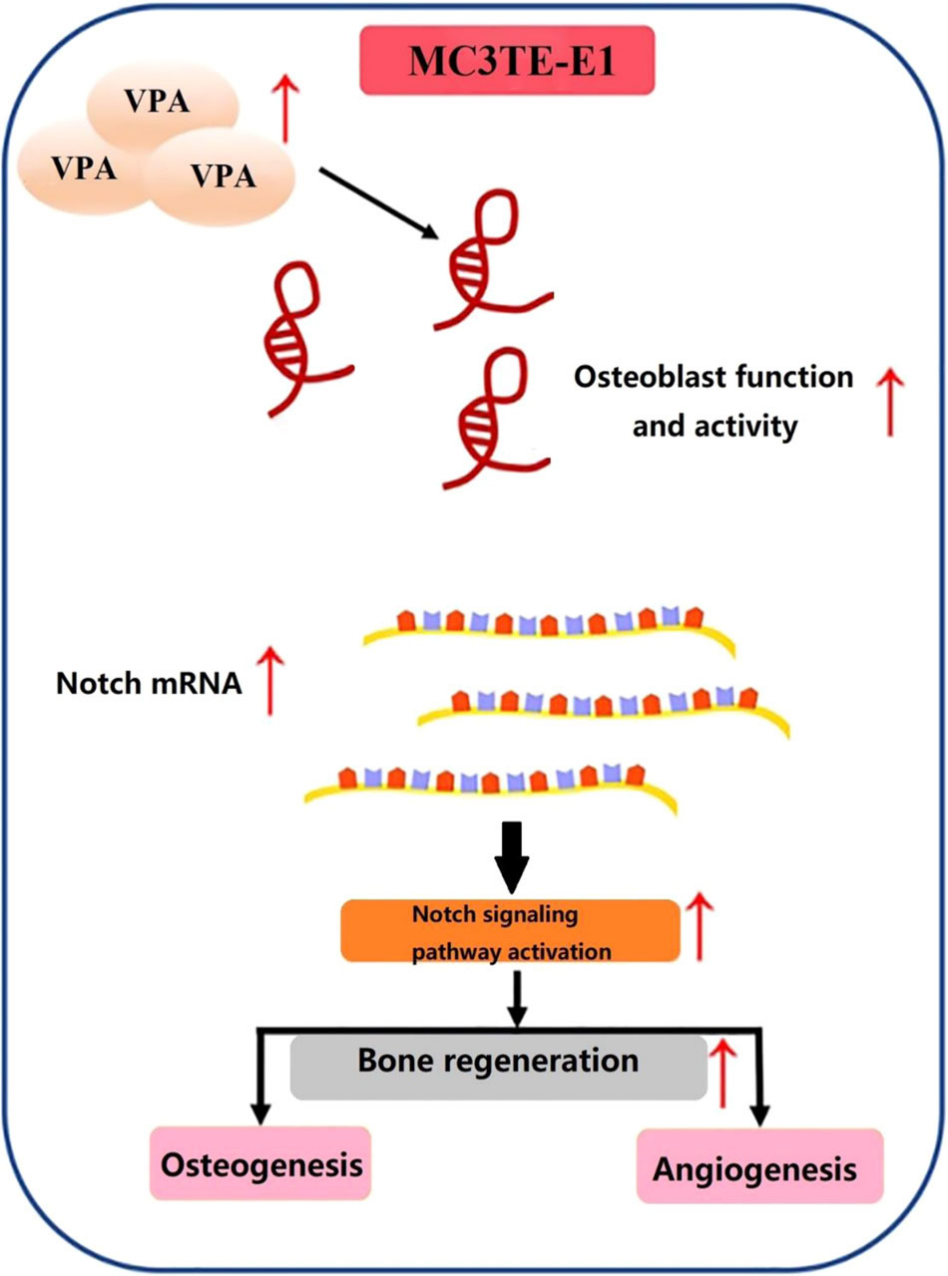

## Introduction

Osteoporosis is a health-concern metabolic bone skeletal disease characterized by increased bone loss and bone-structure deterioration, which will lead to reduced bone mineral density (BMD) and bone strength and increased risk of fragility fractures [1–3]. Most osteoporotic fractures and defects occur in low-energy trauma in elderly people with femoral neck, distal radius and vertebral fractures [4]. In the event of a bone fracture or defects, bone tissue has the unique regeneration ability to replace damaged tissue by constant remodeling with osteoclasts, osteoblasts, osteocytes, and bone-lining cells. When bone defect reaches a certain volume, it is difficult to complete bone-defect healing by its regeneration ability, especially combined with osteoporosis characterized by markedly impaired bone-repair ability [5, 6]. Over the last few decades, bone-substitute materials have received significant attention, and numerous bone biomaterials researches have been reported [7, 8]. While autologous bone graft is still considered to be the “Gold Standard” for bone-defect reconstruction, the complications such as extension of surgical time, increase of surgical sites, and infection chance and aggravation of patient pain may therefore promote people to find and design new alternative drugs and biological materials [9].

Valproic acid (VPA), a branched-chain fatty acid extracted from Valeriana officinalis, which has been widely used in people with epilepsy for more than 20 years owing to its remarkable effect and 80% oral bioavailability [10]. VPA is an inhibitor of the CYP450 enzyme characterized by minimal effects on hepatic metabolic enzymes that has been used as an antiepileptic drug for many years [11]. Previous studies have reported that VPA functions as a histone deacetylase inhibitor (HDACi), with the specific inhibiting activity by binding to the catalytic center of HDACs, which stimulate apoptosis and inhibit the proliferation of cancer cells [12]. In vitro experimental studies have reported that VPA regulates cell histone acetylation to accelerate osteoblast differentiation and the maturation processes [13, 14]. Interestingly, several cellular and our previous animal experiment have shown VPA’s beneficial effects on bone health [15–18].

Although our study has confirmed that VPA plays a positive effect in the process of bone remodeling [19], the local administration of VPA in the treatment of osteoporotic bone defects is lacking and limited. Based on these previous studies and our previous animal experiment, we hypothesized that local administration of VPA may have a positive effect on bone-defect regeneration in an OVX rat model. The aim of the present study was to investigate the effect of local treatment with VPA on bone defect in an OVX rat model, and preliminary exploration of possible mechanisms.

## Materials and methods

### Experimental animal

The current study employed 40 healthy female SD rats (12 weeks of age, weighing 230 ± 25 g). All animals were housed in groups of five in cages in a temperature- controlled environment (25 ± 1 °C, 55–65% relative humidity; 12 h of artificial lighting) in the central laboratory of Yijishan hospital. All experimental rats were kept on pellet feed with standard laboratory diet and tap water, ad libitum. All surgical procedures and drug treatment during the course of the experiment, as well as the sacrifice of the rats at the end of the experiment were approved by the Animal Research Committee.

### Preparation of VPA hydrogel scaffolds

The VPA hydrogel was synthesized as described [20]. Briefly, 200 μl of poloxamer 407 hydrogel (BASF, Ludwigshafen, Germany) (1/4 volume ratio) was mixed with 50 μg of valproic acid and 0.01 M phosphate-buffered saline (PBS) (pH 7.4, at 4 °C). The VPA hydrogel solutions were introduced in Teflon molds (1.5-mm diameter × 4.0-mm height), and samples were immersed in 0.9 wt.% of NaCl solution at 37 °C for setting.

### Animal experiments

In order to establish an osteoporosis model, the rats were subjected to bilateral ovariectomy(OVX, *n* = 25) or sham operation (Sham, *n* = 15) according to a previously described protocol [21, 22] and were kept for 12 weeks. Subsequently, each of five rats from OVX group and Sham group were randomly selected and executed. Bilateral femora were collected and measured by Micro-CT and HE staining to verify the establishment of standard postmenopausal osteoporotic animal models. Then all animals were randomly divided into three groups of 10 rats each: Sham group, OVX group, and VPA-treatment group (VPA group). Once osteoporosis was confirmed, a drilling bone defect with 1.5-mm external diameter of the anteroposterior channel was created by an electric motor with a speed of 1500 rpm in the femoral condyle of the remaining rats according to our previous reports [19, 23]. The rats were classified to VPA group and were implanted and treated with VPA hydrogel. Two intraperitoneal injections of calcein(20 mg/kg) were injected on the 3rd and 10th day before the rats were sacrificed. After 12 weeks of treatment, the rats undergoing bone defect surgery were sacrificed using an overdose of chloral hydrate. Femur samples were harvested. Femurs were fixed at 4 °C with 4% paraformaldehyde.

### Cell culture and alkaline phosphatase (ALP) staining and Alizarin Red (RES) staining

As an osteoblast precursor cell line, MC3TE‐E1 was obtained from the Institute of Biochemistry and Cell Biology, CAS (Shanghai, China). MC3TE‐E1 was cultured in 24-well plates at 1 × 10^4^ cells per well with growth-culture medium. After culturing for 24 h, MC3TE‐E1 cells were plated at a density of 1 × 10^4^ cells/ml in 24-well plates and cultured in growth medium supplemented with 10^−8^ M dexamethasone (Sigma), 50 μg/ml ascorbic acid (Sigma), and 5 mM β-glycerol phosphate (Sigma). Then the medium was added with phosphate-buffered saline (PBS) or valproic acid (10^−6^ M). The medium was changed every four days during osteogenic differentiation. After induction for 14 and 21 days, osteogenesis was evaluated by staining MC3T3-E1 osteoblasts with ALP substrate mixture (ALP staining kit, Sigma) and Alizarin Red reagent (RES, Cyagen Biosciences, Guangzhou, China) as protocol described, respectively.

### Micro-CT evaluation

Formation of new bone in defect areas was evaluated by Micro-CT (Bruker Skyscan 1272 system, Kontich, Belgium). The parameter is set to 55 kV and 114 m A with a thickness of 0.048 mm per slice in medium-resolution mode, 1024 reconstruction matrix, and 200 ms integration time. These images and parameters of trabecular bone with a distance of 1 mm proximal from the end of the growth plate in femoral metaphysis were compared between the Sham group and OVX group to confirm the osteoporosis rat model. For evaluation of bone formation in the defect area, a 1.5-mm-diameter area in the center of each bone defect was selected as the volume of interest (VOI). After 3D reconstruction, bone mineral density (BMD), bone mineral content (BMC), bone volume fraction (BV/TV), trabecular number(Tb.N), trabecular thickness (Tb.Th), and trabecular separation(Tb.Sp) were automatically determined for identification of osteoporosis model, while BMD, BV/TV, Tb. N, Tb. Th, Tb. Sp, and the mean connective density (Conn. D) in VOI regions were used to evaluate new bone formation, using a protocol provided by the manufacturer of the Micro-CT scanner as previously described [24, 25].

### Biomechanical examination

Compression testing of bone samples was performed immediately after the micro-CT scan. The distal femoral metaphysis of each femur was placed in a 5 mm-wide and 2 mm-deep notch of an aluminum alloy base, which was fixed to the mechanical testing system(Instron 5566; Instron, Norwood, MA, USA). The compression load was applied to the ventral aspect of the condyles at 2 mm/min until failure. Ultimate load (N) was calculated from the load-deformation curve.

### Histomorphometric analysis and immunofluorescence staining

Part of the femora was decalcified in 10% EDTA (pH 7.4) for 4 weeks and then embedded in paraffin. Four-micrometer-thick longitudinally oriented along the defect sections were used for staining. The specimens were stained with hematoxylin and eosin (H&E) according to a standard protocol, viewed under a light microscope and the stained areas were quantified using a BI-2000 medical image analysis system (Chengdu TME Technology Co, Ltd., Chengdu, China). Vascular endothelial growth factor (VEGFA) and recombinant human bone morphogenetic protein-2 (BMP-2) staining were used to quantify the expression of osteogenesis and vascularization factors in the defect area. In brief, fresh bone sections were stained with individual primary antibodies to rats VEGFA (Abcam, ab206887, 1:100) and BMP-2 (Abcam, ab214821, 1:100), overnight at 4 °C. Subsequently, the secondary antibodies conjugated with fluorescence (Jackson Immuno Research, 415-605-166, 1:500; 315-605-003, 1:250) were used at room temperature for 1 h while avoiding light and observed under a confocal microscope (FLUOVIEW FV300, Olympus). Calcein double labeling in undecalcified bone slices was observed under a fluorescence microscope (FLUOVIEW FV300, Olympus) to quantify bone mineralization in the defect area.

### Western blot analysis

MC3TE-E1 cells 3 days after drug intervention were prepared for Western blotting as previously described. Bone tissue in the defect area was processed with liquid nitrogen. After that, spin-OUT columns (GT1200, G-Biosciences, St Louis, USA) were used for the rapid purification of protein. The membrane was incubated with Anti-alkaline phosphatase (ALP, Abcam, ab198554, 1:1000), Anti-RUNX family transcription factor 2 (RUNX 2, Abcam, ab236639, 1:1000), Anti-osteopontin (OPN, Abcam, ab214050, 1:1000), Anti-Osteocalcin (OC, Abcam, ab133612, 1:1000), Anti-VEGFA (Abcam, ab214424, 1:1000), Anti-BMP-2 (Abcam, ab214821, 1:1000), Anti-Notch1 (Abcam,ab52627, 1:1000), Anti-HEY1 (Abcam, ab154077, 1:1000), Anti-Jag1 (Abcam, ab109536, 1:1000) and Anti-HES1 (Abcam, ab119776, 1:1000) overnight at 4 °C. Protein expression levels were normalized to Glyceraldehyde 3 phosphate dehydrogenase (GAPDH; Boster, Wuhan, China, 1:2000) protein levels. The next day, the membranes were washed and incubated with the corresponding secondary antibody, diluted at 1:1000 for 2 h at room temperature. The membrane was incubated with ECL-enhanced claim inesence solution and then exposed to X-ray films (Pierce Biotechnology Inc., Rockford, IL).

### Reverse transcription and real-time polymerase-chain reaction (RT-PCR) analysis

According to the manufacturer’s instructions, total messenger RNA (mRNA) was extracted using the total RNA extraction kit (Takara, Kusatsu, Japan). Complementary DNA (cDNA) was obtained from total RNA using first Strand cDNA Synthesis Kit (Toyobo, Osaka, Japan). Then synthetic cDNAs and specific primers were used for qRT PCR with the TB GreenTM Premix Ex Taq II (Tli RNaseH Plus) kit (Takara, Kusatsu, Japan) on the CFX ConnectTM Real-Time System (Bio-Rad, Singapore). GAPDH was used as an internal control. Sequences of primers for the reference gene (GAPDH) and interested genes are listed in Table 1.

**Table 1:** Nucleotide sequences for real-time RT-PCR primers

Genes	Forward (5′-3′)	Reverse (5′-3′)	Product length
Notch 1	CGGGGCTAACAAAGATATGC	CACCTTGGCGGTCTCGTA	68
HES1	GGAAATGACAGTGAAGCACCT	CAGCACACTTGGGTCTGTG	78
HEY1	GGCAGGAGGGAAAGGTTACT	CTCAGATAACGCGCAACTTC	79
JAG1	GGCAACACCTTCAACCTCA	GCCTCCACAAGCAACGTATAG	103
GAPDH	TGCGATGGGTGTGAACCACGAGAA	GAGCCCTTCCACAATGCCAAAGTT	130

### Statistical analysis

All data are shown as mean ± standard deviation, analyzed using SPSS 19.0 software(IBM SPSS Statistics for Windows, Armonk, NY, USA). One-way analysis of variance(ANOVA) was used for multiple between-group comparisons followed by Tukey’s post hoc test. Paired-samples t test was used for comparisons of normal groups and OVX groups. A value of *P* ≤ 0.05 was considered to reflect significance.

## Result

### Cell function and related protein expression

**Fig. 1:**
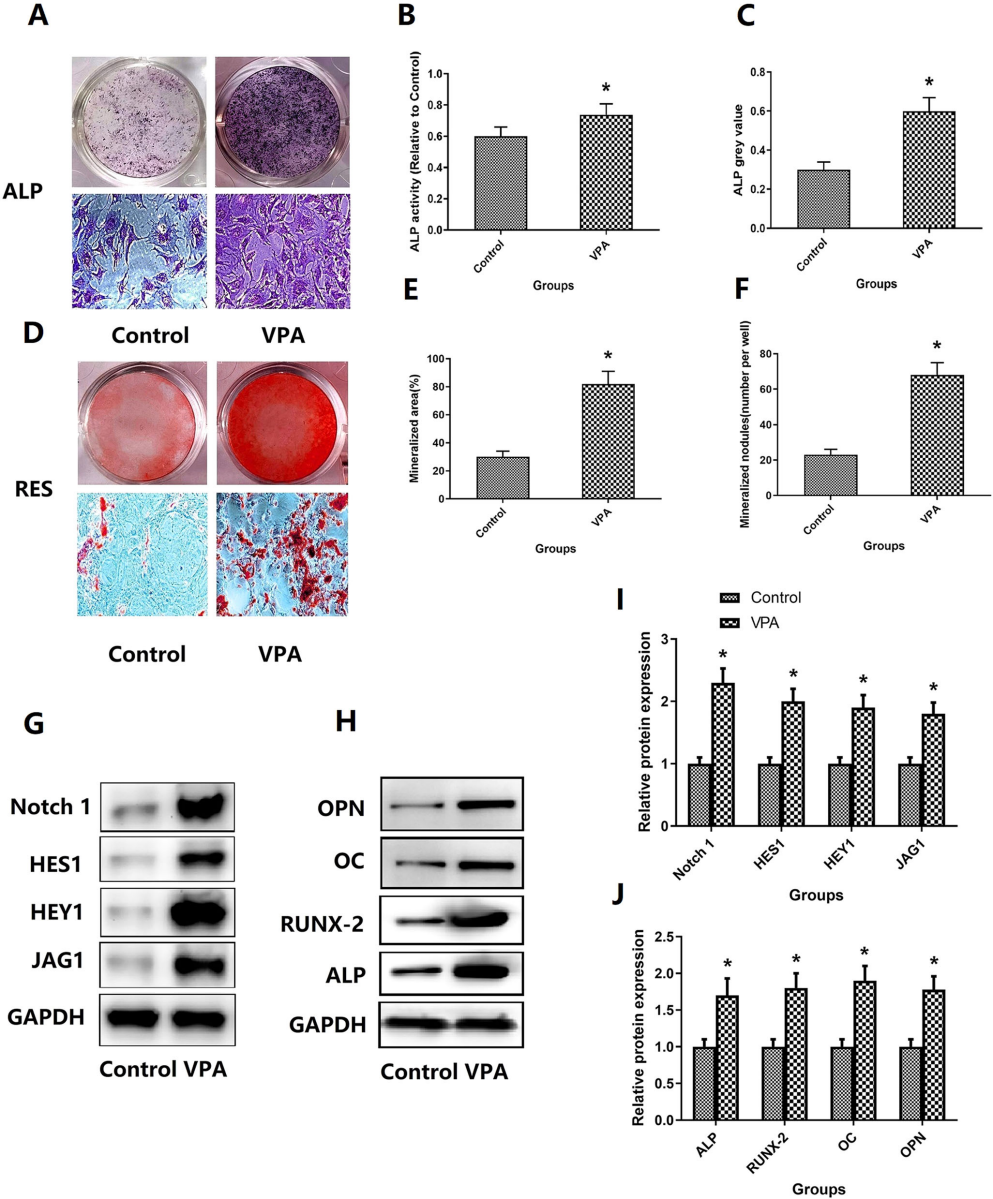
VPA treatments affect cell function and related protein expression. **A**, **D** Representative pictures of ALP staining and Alizarin red staining of osteoblasts after VPA intervention. **B**, **C**, **E** and **F** The quantification of mineralized nodules, mineralized area, ALP activity, ALP gray value. **G**, **H**, **I**, **G** and **J** The relative expression levels of osteoblast-related proteins after VPA intervention and representative pictures of WB detection. *Vs. Control group, *p* < 0.05

In order to determine the effect of VPA on MC3T3-E1 cells function and related protein expression, this study further conducted ALP staining, RES staining and WB analysis. As shown in Fig. 1A, the ALP staining and RES staining with quantification of area in osteogenic differentiation of MC3T3-E1 cells is shown in Fig. 1B. The mineralized nodules (number per well), mineralized area (%), ALP activity and ALP gray value of VPA group were significantly higher than that of control group (*P* < 0.05). The osteogenic protein expressions including ALP, OPN, RUNX-2, and OC of VPA group were significantly higher than that of the control group (*P* < 0.05). These results indicate that the treatment with VPA can significantly increase MC3T3-E1 cell function and related protein expression.

### Osteoporosis animal model validation

**Fig. 2:**
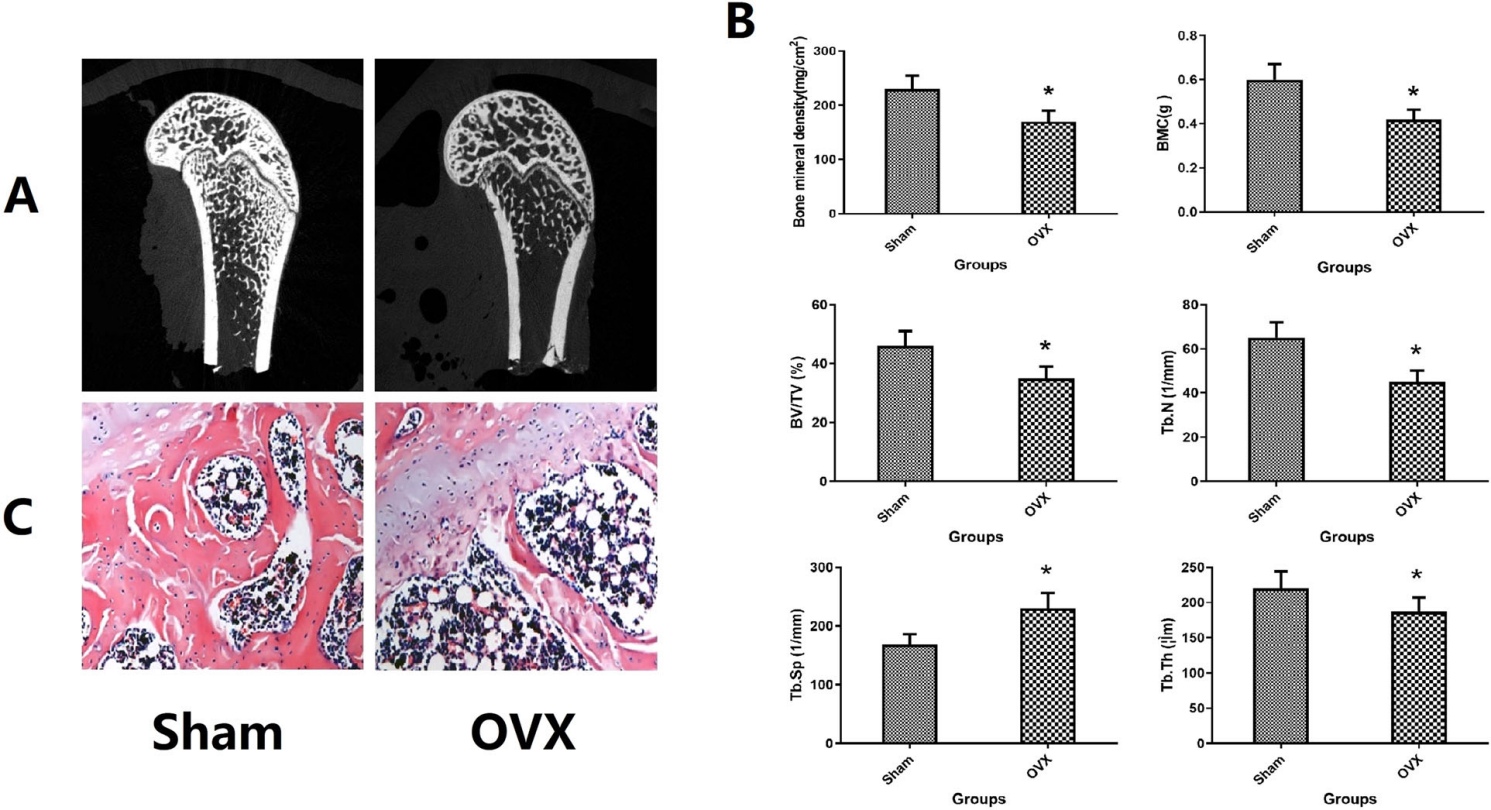
**A** 2D micro-CT images of femoral metaphysis in sham and OVX rats, the scale bar represents 1 mm. **B** The BMD, BMC, BV/TV, Tb.N, Tb.Th, and Tb.Sp of trabecular bone of femoral metaphysis in OVX group and sham group. **C** Representative H&E staining for the normal and osteoporotic femur(magnification of 20). *P < 0.05 versus Sham group

A total of 6 rats died during the experiment, including anesthetic accidents, infection and surgical accidents. No animal death was found in the first operation. The death of rats occurred during or after the second operation, including OVX group (*n* = 2), Sham group (*n* = 2), and VPA group (*n* = 2). After 12 weeks of ovariectomy and sham operation, the femurs of 5 rats were randomly selected and observed by Micro-CT and HE staining microscope as shown in Fig. 2. Imaging and tissue sections clearly show that there is a serious trabecular loss in the metaphysis of the femur in the OVX group; the quantitative results of Micro-CT include BMD, BMC, BV/TV, Tb. Th, Tb. N, and Tb. Sp, which shows that there are significant statistical differences in the above-mentioned indexes between the two groups (*P* < 0.05). These results indicate that the osteoporotic rat model induced by ovariectomized surgery in our experiment achieves the expectation.

### Micro-CT evaluation

**Fig. 3:**
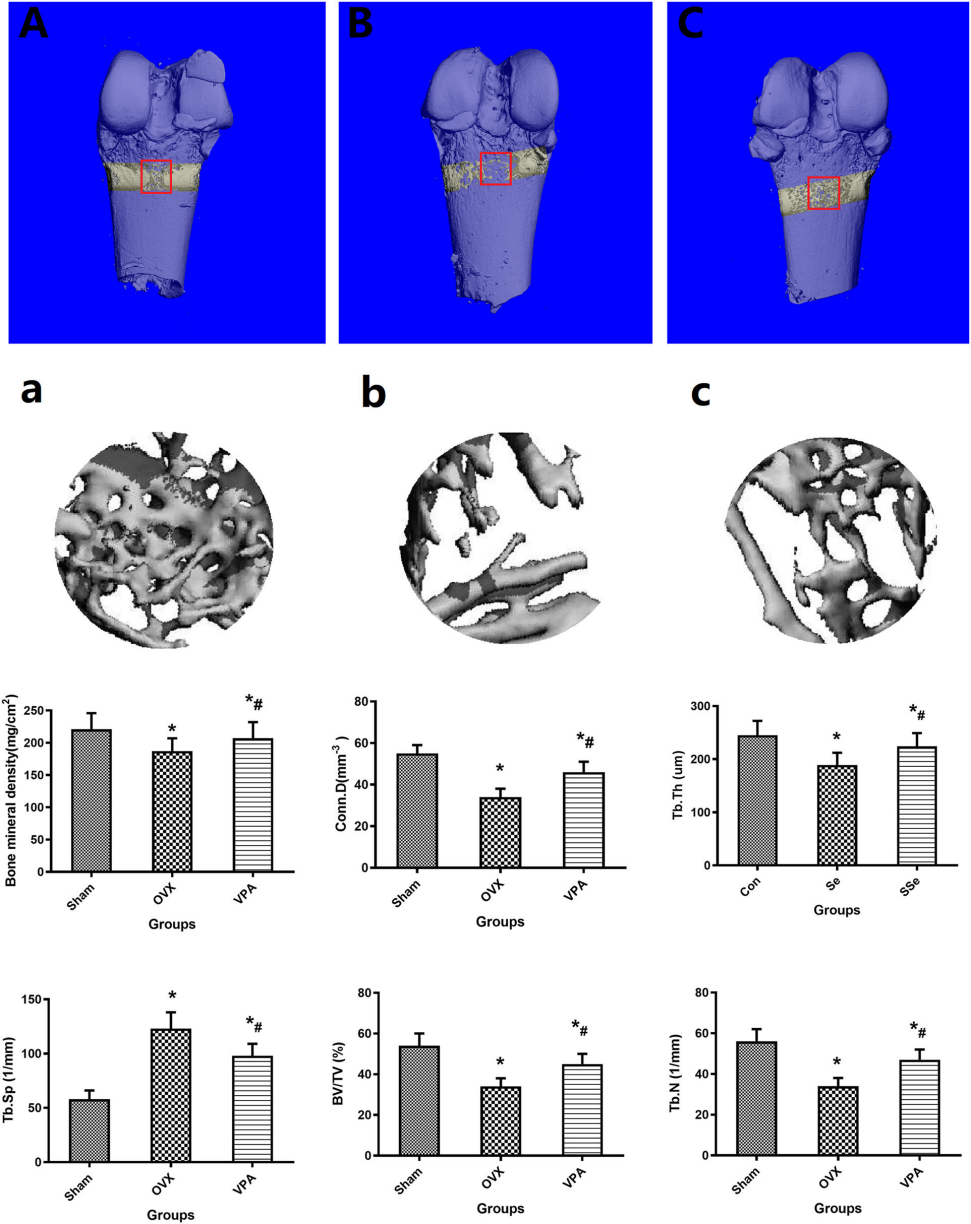
The representative pictures of Micro-CT images of the distal femur 12 weeks after biomaterials implanted from group of Sham (**A**, **a**), OVX (**B**, **b**), and VPA (**C**, **c**). The scale bar represents 2 mm. Quantitative results of new trabeculae bone in defect area including BMD, BV/TV, Tb. N, Conn.D, Tb. Th, and Tb.Sp. *Vs. Sham group, *p* < 0.05, ^#^Vs. OVX, *p* < 0.05

The 3D reconstruction images and middle part (Fig. 3A–C, a–c) of Micro-CT clearly shows us the bone remodeling of the defect area after 12 weeks of treatment with different intervention methods. As we expected, the defect area of the Sham group was almost filled with bone tissue, while large amounts of bone tissue were found in the VPA group, but it was difficult to find the bone tissue in the OVX group. The quantitative results were expressed as BMD, BV/TV, Tb. Th, Tb. N, Conn. D, and Tb. Sp (Fig. 3). Therapy with VPA showed positive effects on all micro-CT parameters. Compared with group OVX, local treatment with VPS shows better bone microscopic parameters, including the highest BMD, BV/TV, Tb. N, Conn.D, Tb. Th, and a lower Tb. Sp (*P* < 0.05).

### Biomechanical testing, histological and fluorescent analysis

**Fig. 4:**
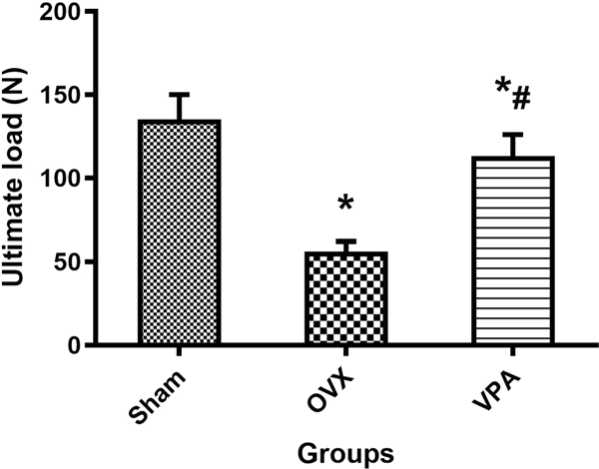
Biomechanical testing results after VPA treatment. *Vs. Sham group, *p* < 0.05, ^#^Vs. OVX, *p* < 0.05

**Fig. 5:**
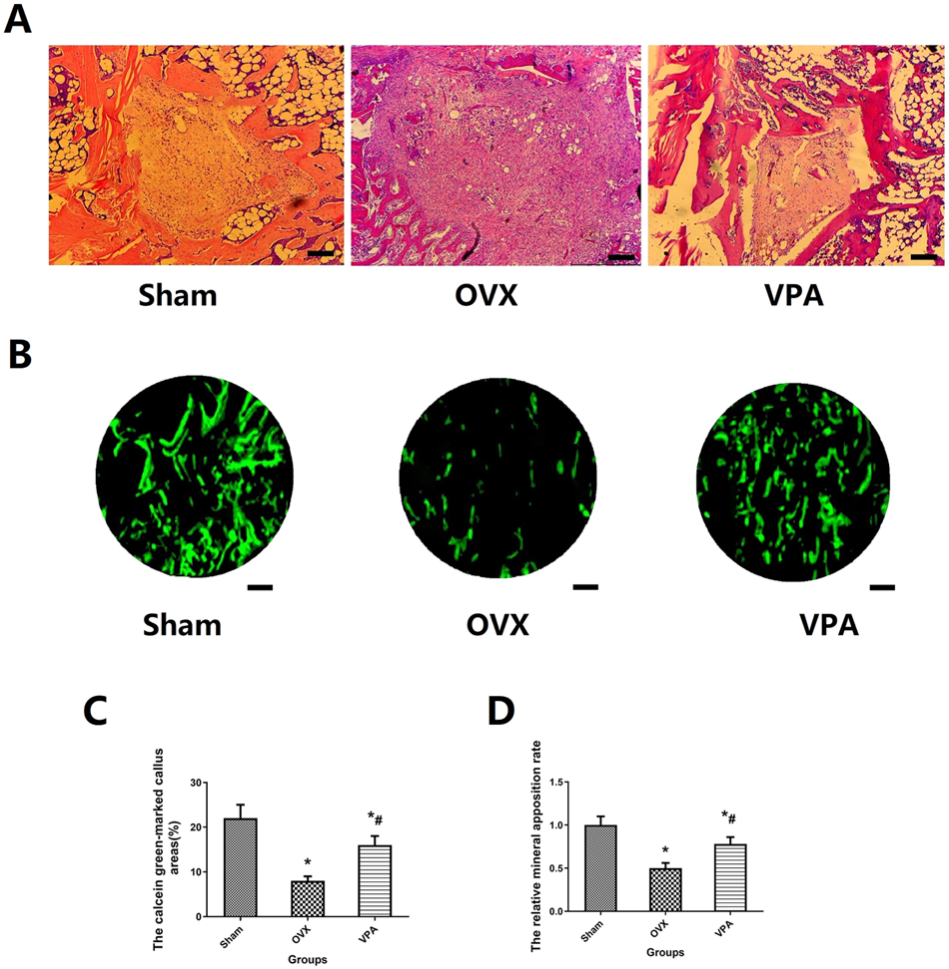
Bone regeneration of defected area by histological (**A**, magnification, ×10) and fluorescent analysis (**B**, magnification, ×200). **C**, **D** Total fluorescently marked defect area (%) and relative bone mineralization (green/green marked defect area) after treatment. *Vs. Sham group, *p* < 0.05, ^#^Vs. OVX, *p* < 0.05

Biomechanical testing, histological, and fluorescent images showing bone repair in defect for different treatments, as shown in Figs. 4 and 5. In 12 weeks, a large amount of bone tissue fills the defect area in the Sham group and VPA group. In the OVX group, only a very small amount of new bone tissue can be observed, and large defect areas still exist. The biomechanical test results show that the ultimate strength of the VPA group is significantly higher than that of the OVX group (*p* < 0.05). In fluorescent analysis, local treatment with VPA showed the larger calcein green-marked defect area (*p* < 0.05), and VPA treatment exhibited the higher values of relative bone mineralization (green/green-marked defect area) (*p* < 0.05), compared to that of the OVX group.

### Immunofluorescence and RT-PCR, WB analysis of osteogenesis and angiogenesis-regulatory factors

**Fig. 6:**
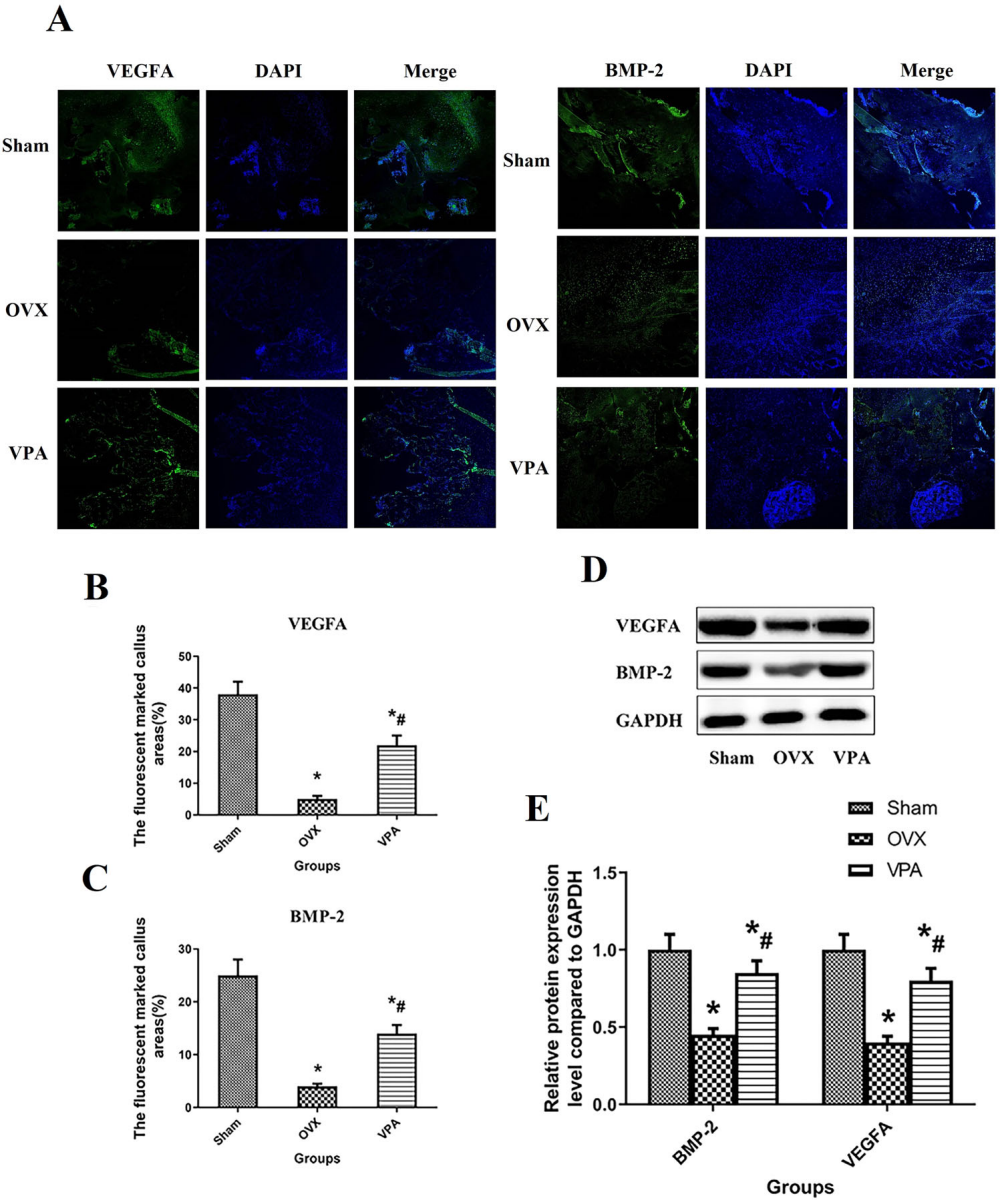
After VPA intervention, the relative expression levels of VEGFA and BMP-2 in the bone defect area were detected by immunofluorescence and the representative pictures of WB detection. **A** VEGFA and BMP-2 expression measured by immunofluorescence; **B**, **C** immunofluorescence was used to detect the quantitative results of VEGFA and BMP-2 expression in the tissues of the bone-defect area; **D** the representative pictures of WB detection; **E** the quantitative results of VEGFA and BMP-2 expression in the tissues of the bone-defect area. *Vs. Sham group, *p* < 0.05, ^#^Vs. OVX, *p* < 0.05

**Fig. 7:**
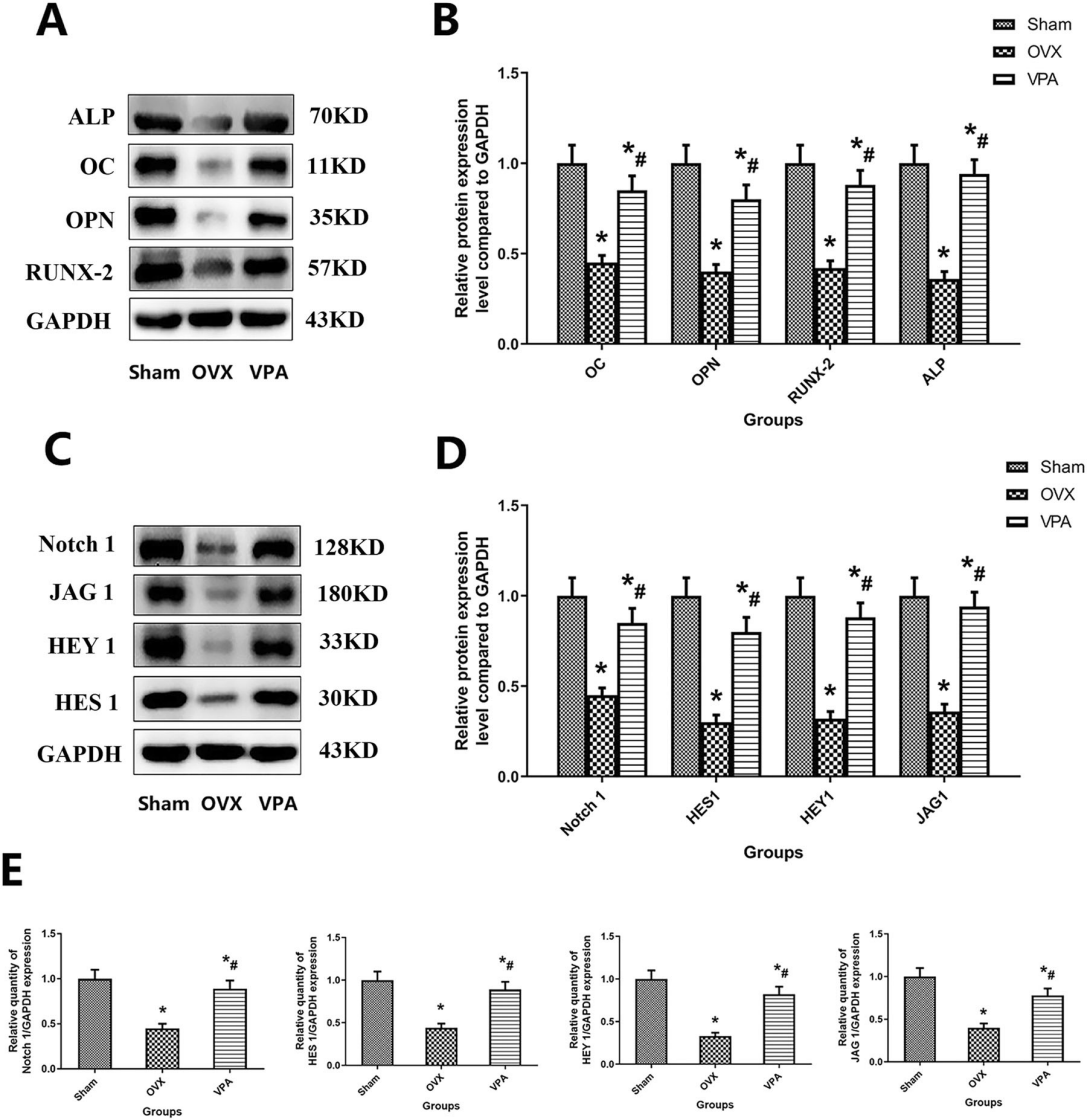
Protein and gene expression of defect area bone tissue after different treatment. **A**, **B** WB detection results and quantitative detection results of osteoblast-related regulatory protein expression. **C**, **D** WB detection results and quantitative detection results of specific protein expression of Notch pathway. **E** The quantitative detection results of specific gene expression of Notch pathway. *Vs. Sham group, *p* < 0.05, ^#^Vs. OVX, *p* < 0.05

The osteogenesis and angiogenesis regulator of bone defect measured by immunofluorescence and WB clearly show us the expression of VEGFA and BMP-2 of the defect area after 12 weeks of treatment with different intervention methods (Figs. 6 and 7). As we expected, the defect area of the VPA group was almost filled with immunofluorescence for VEGFA and BMP-2, but it was difficult to find immunofluorescence in the OVX group.

The quantitative results measured by WB were expressed as Notch 1, HES1, HEY1, JAG1, OC, OPN, RUNX-2, ALP, VEGFA and BMP-2. Therapy with VPA showed positive effects on Notch 1, HES1, HEY1, JAG1, OC, OPN, RUNX-2, ALP, VEGFA and BMP-2 expression. Compared with group OVX, local treatment with VPA shows the higher protein expression with Notch 1, HES1, HEY1, JAG1, OC, OPN, RUNX-2, ALP, VEGFA, and BMP-2 (*P* < 0.05).

Gene expression of defect area bone tissue after different treatment, as shown in Fig. 7. At 12 weeks, the VPA group showed increased Notch 1, HES1, HEY1, and JAG1 than the OVX group (*p* < 0.05). These results indicate that the Notch pathway of VPA treatment is activated, and the expression of Notch 1, HES1, HEY1, and JAG1 is upregulated.

## Discussion

In this experimental study, a standard osteoporotic animal model was established 12 weeks after bilateral ovariectomy. Bone-forming capacity was evaluated with local administration with VPA in OVX rats for 12 weeks after distal femur-defect creation. The current study provides evidence, by biological activities of MC3T3-E1 osteoblasts, Micro-CT, Western blot, real-time PCR, biomechanical testing, histological, and immunofluorescence analyses, confirming the positive effects on bone of local VPA therapy in OVX rats. In addition, the harmful effects on bone regeneration after bone injury were more obvious in OVX rats compared with Sham group rats. After local treatment with VPA, osteogenic ability and bone microstructure parameters of defects were significantly improved in OVX rats. Therefore, our findings indicated that VPA reversed the effects of estrogen deficiency on repair, strengthened the bone-regeneration capacity, and enhanced the repair of femoral metaphyseal defects in OVX rats.

In this study, we used standard bilateral OVX rats, the most frequently used hormone-deficient osteoporosis animal model [26], to mimic osteoporosis observed in menopausal women, and then a hydrogel containing VPA implanted into the femoral condyle defect under the state of osteoporosis to simulate the state of bone defect in the human body. Interestingly, a similar phenomenon that the femoral condyle defect in OVX rats provide a poor bone regeneration with osteoporotic bone was also reported in our previous studies [27, 28]. However, osteoporosis stimulation decreased bone formation and bone repair when compared with the Sham group. These results above confirm that the bone regeneration of bone defects is negatively influenced by osteoporotic bone conditions. Therefore, in order to increase bone-formation potential further, it is necessary to increase bone-regeneration capacity in the state of osteoporosis.

Hydrogel, a kind of three-dimensional, insoluble hydrophilic polymer with good biocompatibility, biodegradability, large water content, and tissue-like flexibility, is used to form drug-delivery vehicles [29]. This material is able to transport nutrients and metabolites from the extracellular matrix for its special structure, which contributes to cell proliferation and differentiation [30], and leads to its wide use in tissue engineering. Currently, a vast array of hydrogels have been investigated, including alginate, fibrin, chitosan, hyaluronic acid, and gelatin. Previous research has confirmed that poloxamer 407 hydrogel is an effective controlled delivery system, and can be competent as a local drug carrier [31]. Therefore, poloxamer 407 hydrogel was used as an effective carrier for VPA in this study. In the study, local treatment with hydrogel containing VPA produced anabolic effects on new bone in femoral condyle defect. Recent studies have shown interesting results for this drug in bone metabolism and bone remodeling, suggesting that this medication might be relevant for the treatment of osteoporosis, since it has been shown to be effective in preventing bone loss [15–17]. Moreover, recent studies have shown the role of VPA in pathogenesis, tissue regeneration, and can serve as a useful Notch pathway activator and that it is a potential alternative drug for regulation of cell histone acetylation [17]. Indeed, a study has shown that this drug reduces local tissue damage in glucocorticoid–induced osteonecrosis of the femoral head [15]. Previous research has confirmed that intermittent treatment with VPA resulted in significant increases in trabecular thickness and trabecular number while decreasing trabecular separation, which improved the extrinsic biomechanical properties of bone, prevention of bone fragility, and decreased fracture risk. In vitro cell research has been conducted to evaluate the effect of VPA on biological function of osteoblasts and osteoclasts [16]. Our current results showed that local treatment with VPA increased bone repair and enhanced bone regeneration for 12 weeks.

Why did local treatment with VPA show a positive effect on bone formation in the defect area? Therefore, further investigations are required to confirm these findings and explore possible mechanisms for the observed association. In order to further investigate the potential mechanisms, we also performed bone-tissue immunofluorescence, qRT-PCR, and western blotting experiments to analyze the mRNA expression of related gene, regulatory factor, and protein contents. Recently, studies indicated that notch signaling also plays a vital role in mineralization of bone tissue via a direct regulation effect on osteoblastic activity [32, 33]. Besides, a large body and animal of emerging evidence has proved that angiogenesis plays a key role in bone repair [34, 35]. Similarly, angiogenesis and osteogenesis were regulated by a variety of growth factors, such as VEGFA and BMP-2 [36, 37]. In this study, the influences of VPA on function of osteogenic biological MC3T3-E1 cells were further detected. As we hypothesized, VPA can significantly promote ALP expression and mineralization in MC3T3-E1 osteoblasts as assessed by ALP staining and Alizarin red staining. The results of WB and RT-PCR show that the dose of VPA used in this study can significantly promote the expression of cellular osteogenic regulatory proteins, such as ALP, RUNX-2, OPN, BMP-2, and OC. In addition, it can activate the Notch pathway and significantly upregulate Notch 1, HES1, HEY1, and JAG1 in MC3T3-E1 osteoblasts and bone tissues. Bone-tissue immunofluorescence and WB clearly showed us the expression of VEGFA and BMP-2 in bone tissue in the defect area, which further confirmed that local blood-vessel formation and bone formation were significantly improved after VPA intervention. Combining the above results, what we can explain is local treatment with VPA can markedly promote osteogenesis and angiogenesis, which causes acceleration of local bone formation and enhanced mineralization ability, and resulting in achieved finally bone regeneration and improved bone strength.

As far as we know, this is the first study of the effect of local administration with VPA on the regeneration of bone defect under osteoporotic conditions. Nevertheless, this study had several deficiencies. The mechanisms underlying the effects of VPA on osteogenic differentiation of MSCs should be elucidated. Delivery systems that allow the sustained release of VPA should be developed for effective bone regeneration in vivo. The optimal dosage of VPA should be determined for bone regeneration by using animal studies.

## Conclusion

In summary, our study suggests that the treatment solution with local administration with VPA is useful to improve the initial bone regeneration of defects by increasing bone formation and angiogenesis in osteoporotic rats. Besides, this benefit effect may be mediated by locals used with VPA via notch signaling pathway.
